# Rheumatism

**Published:** 1887-04

**Authors:** 


### RHEUMATISM.

He who gives to the public an effectual cure for so common and crippling a disease as rheumatism is a benefactor to his race. Our correspondent assures us that the following formula, which was obtained from a distinguished physician of extensive practice, has been prescribed by him in no less than twenty-four cases of *acute rheumatism*, and in every instance effected a radical cure.

RECIPE.

Ferri et Quinia Cit.......................................3 drachms.
Potassa Iodide.............................................4 "
Vin Colch Rad, }
Tr. Cimieifuga, } ......................................each 1½ ounces.
Tr. Hyoscyamia, }
Fluid Ext. Stillingia Comp...................................1 ounce.
Aqua sufficient with foregoing to fill 8 ounce bottle.

Dose, two teaspoonfuls in water three times a day.

Should any of our readers using the above prescription, find it efficacious, we would very much like to be advised of it. It might suggest the propriety of subscribing for HALL'S JOURNAL.

SIMPLE WATER TESTS.—Test for hard or soft water—Dissolve a small quantity of good soap in alcohol. Let a few drops fall into a glass of water. If it turns milky, it is hard ; if not, it is soft. Test for Earthy Matters or Alkali—Take litmus paper dipped in vinegar, and if, on immersion, the paper returns to its true shade, the water does not contain earthy matters or alkali. If a few drops of syrup be added to a water containing an earthy matter, it will turn green. Test for Carbonic Acid—Take equal parts of water and clear lime water. If combined or free carbonic acid is present, a precipitate is seen, to which, if a few drops of muriatic acid be added, an effervescence commences. Test for Magnesia—Boil the water to a twentieth part of its weight, and then drop a few grains of neutral carbonate of ammonia into a glass of it, and a few drops of phosphate of soda. If magnesia be present, it will fall to the bottom. Test for Iron—1. Boil a little nut gall and add to the water. If it turns gray or slate, black iron is present. 2. Dissolve a little prussiate of potash, and, if iron is present, it will turn blue. Test for Lime—Into a glass of water put two drops of oxalic acid and blow upon it. If it gets milky, lime is present. Test for Acid—Take a piece of litmus paper. If it turns red, there must be acid. If it precipitates on adding lime water, it is carbonic acid. If a blue sugar paper is turned red, it is a mineral acid.—*Scientific American.*

